# Musculoskeletal pains among amateur and professional athletes of five disciplines in Senegal: a preliminary study

**DOI:** 10.1186/s12891-023-06275-3

**Published:** 2023-03-22

**Authors:** Hassane Malam Moussa Ahmet, Elysée Claude Bika Lele, Wiliam Richard Guessogo, Wiliam Mbang Bian, Jessica Guyot, Ahmadou, Peguy Brice Assomo-Ndemba, Clarisse Noel Ayina, Loick Pradel Kojom Foko, Caroline Dupré, Nathalie Barth, Bienvenu Bongue, Abdoulaye Ba, Abdoulaye Samb, Samuel Honoré Mandengue, Jerson Mekoulou Ndongo

**Affiliations:** 1 Faculty of Health Sciences of the Abdou Moumouni University of Niamey, Niamey, Niger; 2 Physical Activities and Sport Physiology and Medicine Unit, Faculty of Science, University of Douala, Douala, Cameroon; 3 National Institute for Youth and Sports Yaounde, University of Yaounde I, Yaounde, Cameroon; 4 Mines Saint-Etienne, INSERM, U1059 Sainbiose, University Jean Monnet, Saint-Etienne, 42023 France; 5 Department of Physiology, Faculty of Medicine and Biomedical Sciences, University of Yaounde I, Yaounde, Cameroon; 6 Laboratory of Physiology and Functional Explorations, Faculty of Medicine, Pharmacy and Dentistry, University of Cheikh Anta Diop Dakar, Dakar, Senegal

**Keywords:** Musculoskeletal pains, Athletes, Amateurs, Professionals, Senegal

## Abstract

**Background:**

Musculoskeletal pains (MSPs) in sport are cause of poor performances and loss of competition in athletes. The present study aimed at determining the prevalence of MSPs with regard to sport disciplines and athletic status.

**Methods:**

A cross-sectional study was conducted among 320 Senegalese professional and amateur athletes practicing football, basketball, rugby, tennis, athletics, and wrestling. Rates of MSPs in the past year (MSPs-12) and week (MSPs-7d) were assessed using standard questionnaires.

**Results:**

Overall proportions of MSPs-12 and MSPs-7d were 70 and 74.2%, respectively. MSPs-12 were more frequently reported on shoulders (40.6%), neck (37.1%) and hips/thigh (34.4%), while MSPs-7d were predominant on hips/thigh (29.5%), shoulders (25.7%), and upper back (17.2%). Proportions of MSPs-12 and MSPs-7d varied significantly by sport disciplines, with highest values among basketball players. Again, highest MSPs-12 proportions on shoulders (29.7%, *P* = 0.02), wrists/hands (34.6%, *P* = 0.001), (40.2%, *P* = 0.0002), and knees (38.8%, *P* = 0.002) were seen among basketball players. High proportions of MSPs-7d were seen on shoulders (29.6%, *P* = 0.04) for tennis players, wrists/hands (29.4%, *P* = 0.03) for basketball and football players, and hips/thigh (38.8%, *P* < 0.00001) for basketball players. Football players had reduced risk of MSPs-12 by 75% on lower back (OR = 0.25; 95% CI. 0.10—0.63; *P* = *0.003*) and by 72% on knees (OR = 0.28; 95% CI. 0.08—0. 95; *P* = *0.04*). In contrast, tennis players were more at risk of MSPs-12 on shoulders (OR = 3.14; 95% CI. 1.14–8.68; *P* = *0.02*), wrists/hands (OR = 5.18; 95% CI.1.40–11.13; *P* = *0.01*), and hips/thigh (OR = 2.90; 95% CI. 1.1–8.38; *P* = *0.04*). Professionals were protected from MSPs-12 on neck pain with a significant reduction of risk by 61% (OR = 0.39, 95% CI. 0.21–0.75, *P* = *0.03*).

**Conclusion:**

MSPs are a reality among athletes and their risk is modulated by sport disciplines, athletic status and gender.

## Background

Sport in competitions and its challenges are a catalysts of economic development in developing countries [1]. In amateur and professional athletes, training during preparation is essential for national/international competitions. This training also allows for assessing performances, profiling athletic talent, identifying ability to compete, and identifying weakness and factors determining performances [2].

Athletes, during performance optimization phase, face challenges that expose them to musculoskeletal pains (MSPs) which are sometimes associated with injury. MSP is frequently observed in athletes and is often cause of poor biomechanical performances and even loss of competition. It can also lead to significant recovery periods requiring physiotherapy or surgery [3, 4]. MSPs are a common problem and important warning signals of overuse injury in athletes [5, 6].

MSPs are diagnosed on several body parts of athletes including joints, ligaments, tendons, nerves, muscles, and structures that support limbs, neck and back. Causes of MSPs in athletes are multiple with diverse origin such as repetition of same movement, awkward posture, and host related genetic factors [7–9]. In sports medicine, primary and secondary prevention of MSPs and injuries are particularly challenging in sports medicine constitutes one of the most worrying health problems in athletic world because of their high economic costs, withdrawal of athletes from training and competition, and impaired performances [10, 11]. It is known that physical activity and sport may contribute to prevent MSPs via triggering endorphins production which will result in reduction in inflammation and pain [12–14]. Also, previous studies showed that moderate physical exercises were associated with sustained muscular activity and improved function of joints associated [15].

MSPs in athletes are persistent reality in various disciplines such as wrestling [16], handball [17], rugby [18], soccer [19], tennis, track and field [20], and basket-ball [21], and are cause of disability [6, 21–23] and vary by athletic status [24]. In sub-Saharan Africa there is lack of studies on MSPs in various sport disciplines according to athletic status. This preliminary study was therefore conducted to determine the prevalence of MPSs and associated factors in Senegalese athletes with varying athletic status and different disciplines.

## Methods

### Study design and participants

This was a cross-sectional prospective and analytical study conducted for four months (January-April 2021) in Dakar, Senegal. Participants were recruited at professional and amateur clubs, and were participating regularly to national and international competitions of different sport disciplines (football, basket-ball, rugby, tennis, athletics, and traditional wrestling).. These disciplines were chosen given their great popularity in Senegal. We excluded athletes recovering from musculoskeletal trauma, and under rehabilitation or physiotherapy interventions.

### Sampling

The minimum sample size was computed using the Lorentz’s formula: N = p (1-p) z^2^/d^2^, where N is the minimum sample size; p (= 94%) is the prevalence in the last one year of low back pain reported among athletes by Farahbakhsh et al. [23]; z is statistic for the desired confidence level (z = 1.96 for confidence at 95%) and d the accepted margin of error (d = 0.05). Thus, the minimal sample size found was 87 participants. Finally, a total of 320 athletes was included.

### Ethics

Aims and objectives of the study were first explained to administration staff of athlete clubs and coaches. After obtaining administrative authorization, the study was explained to athletes. An informed and signed consent was obtained from each athlete willing to participate in the study, and they were free to withdraw from study at any time. The study was approved by ethics committee of Cheikh Anta Diop University of Dakar, Senegal (015/2021/CER/UCAD), and conducted as per recommendations of the Declaration of Helsinki revised in 1989.

### Data collection

A structured questionnaire was administered to each participant to socio-demographic and anthropometric data (age, gender, history of injuries), and socio-professional information (discipline, number of training session/ week).

#### Anthropometric parameters

Height was measured using a rod graduated to the nearest centimeter while weight was measured using an electronic scale Tanita BC-532 (Tokyo, Japan). Body mass index (BMI) was determined using Quetelet's formula: BMI (Kg.m^−2^) = Weight (kg) / height^2^ (m^2^).

#### Musculoskeletal pains

A modified Nordic questionnaire [24] adapted to athletes was used to determine MSPs prevalence. This questionnaire focuses on occurrence of MSPs on nine body regions (neck, shoulders, elbows, wrists/hands, upper back, lower back, hips/thighs, knees, ankles/feet) during last 12 months or 7 days after a competition or training session. For each body region.

Parameters evaluated were:

- Presence or absence of aches, pains or discomfort during the last 12 months and / or the last seven days,
- Bad performance due to joint pains,
- Absenteeism during training sessions or competitions during the last 12 months and/or the last seven days owing to pain on at least one body region,
- History of trauma one at least one body region during training sessions or competitions*.*

Therefore:

- A year/12-month MSPs (MSPs-12) was considered present if self-reported ache, pain or discomfort on above mentioned body regions during the last 12 months was reported.
- A week/7-day MSPs (MSPs-7d) was defined as seven-day prevalence of MSPs.
- Pain reported on a former injuries regions was not considered as MSPs.

### Statistical analysis

Quantitative and qualitative variables were expressed as means ± standard deviation and percentage (%) respectively. Unpaired Student's *t*-test was used to compare unpaired quantitative variables. Normality of data was evaluated using Kolmogorov–Smirnov test. The Pearson’s chi-square (χ^2^) test was performed to compare unpaired proportions. Logistic regression models were performed to identify factors associated with MSPs. The association between MSP and independent variables was quantified through computing crude and adjusted odd ratio (cOR and aOR), confidence interval at 95% (95%CI), and level of significance. Statistical analyses were conducted using StatView 5.0 (SAS Institute, Inc., Chicago, USA) software. Statistical significance was set at *P* < 0.05.

## Results

The majority of athletes included in the study were males (69.7%). Football and basket-ball were predominant sport disciplines with proportion of 28.1 and 25%, respectively. More than half participants (64.1%) were amateurs. According to gender, the proportion of males was higher than that of females in all sport disciplines (*P* < *0.0001*). On average, females were older than their male counterparts *(P* < *0.0001).* Anthropometric parameters including height and weight were higher in males compared to females *(P* < *0.0001)* (Table 1). According to athletic status, amateur athletes of the study were more (*P* < 0.0001) into basketball (51.2%), soccer (54.4%), rugby (100%), tennis (100%). On the staturo-ponderal plan, the amateurs were more (*P* = 0.03) height, on the other hand the number of training of the professionals was higher than (*P* < 0.0001) that of the amateurs.

**Table 1:** Gender, socio-demographic and anthropometric characterizations

	**Categories**	n(%)			
**Gender**	Female	97(30.3)			
	Male	223 (69.7)			
			**Gender**		
**Disciplines**		Total n(%)	Female n(%)	Male n(%)	*P*-value
	Basket-ball	80(25)	38(47.5)	42(52.5)	< 0.0001
	Football	90 (28.1)	0(0.0)	90(100)	
	Rugby	58(18.1)	28(48.3)	30(51.7)	
	Wrestling	14 (4.4)	0(0.0)	14(100)	
	Tennis	36(11.3)	12(33.3)	24(66.7)	
	Track and field athletics	42(13.1)	19(45.2)	23(54.8)	
**Athletic status**	Professional	115(35.9)	30(26.1)	85(73.9)	*0.21*
	Amateurs	205(64.1)	67(32.7)	138(67.3)	
Mean ± SD					
**Anthropometric**	Age (years)	25 ± 2	26 ± 1	25 ± 2	< 0.0001
	Height (m)	179 ± 7.6	167 ± 4.5	182 ± 4.2	< 0.0001
	Weight (Kg)	73.2 ± 8.5	67.3 ± 8.3	76.1 ± 13	< 0.0001
	BMI(Kg/m^2^)	22.8 ± 0.9	21.2 ± 2	23.2 ± 0.1	0.9
Training sessions/week		4 ± 1	3 ± 1	4 ± 1	< 0.0001
			**Athletic status**		
			Professional n(%)	Amateur n(%)	*P*-value
	**Gender**	Male	85(38.1)	138(61.9)	0.21
		Female	30(30.9)	67(69.1)	
	**Disciplines**	Basket-ball	39(48.8)	41(51.25)	< 0.0001
		Football	41(45.6)	49(54.4)	
		Rugby	0(0.0)	58(100)	
		Wrestling	14(100)	0(0.0)	
		Tennis	0(0.0)	36(100)	
		Track and field athletics	21(50)	21(50)	
			Mean ± SD		
		Age (years)	25.6 ± 3.7	25.1 ± 3.2	0.14
		Height (m)	1.77 ± 0.88	1.79 ± 0.94	0.03
		Weight (Kg)	74.8 ± 12.8	71.6 ± 12.1	0.13
		BMI(Kg/m^2^)	23.3 ± 4.1	23.9 ± 10.8	0.55
		Training sessions/week	4 ± 1	3 ± 1	< 0.0001

**Fig. 1:**
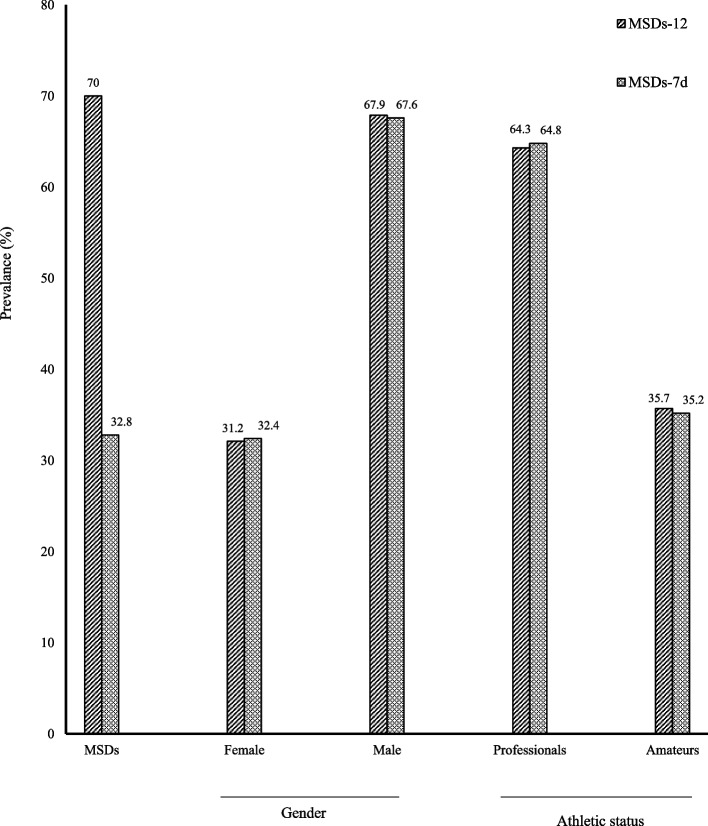
Prevalence of MSPs according to gender and Athletic status. MSPs-12: prevalence of musculoskeletal pains during the 12 last months; MSPs-7d: prevalence of musculoskeletal pains during the seven last days

Proportions of MSPs-12 and MSPs-7d by sport disciplines, athletic status, and gender are illustrated on Fig. 1. Overall proportion of MSPs-12 and MSPs-7d was 70% (95%CI. 64.8–74.8%) and 32.8% (95%CI. 27.9–38.1%), respectively. Also, proportions of MSPs-12 and MSPs-7d were higher in males and professional athletes. For instance, proportion of MSPs-12 was 67.9% in males compared to 31.2% in females (Fig. 1). However, no statistically significant difference was found in MSPs-12 and MSPs-7d proportions with respect to gender and athletic status.

**Fig. 2:**
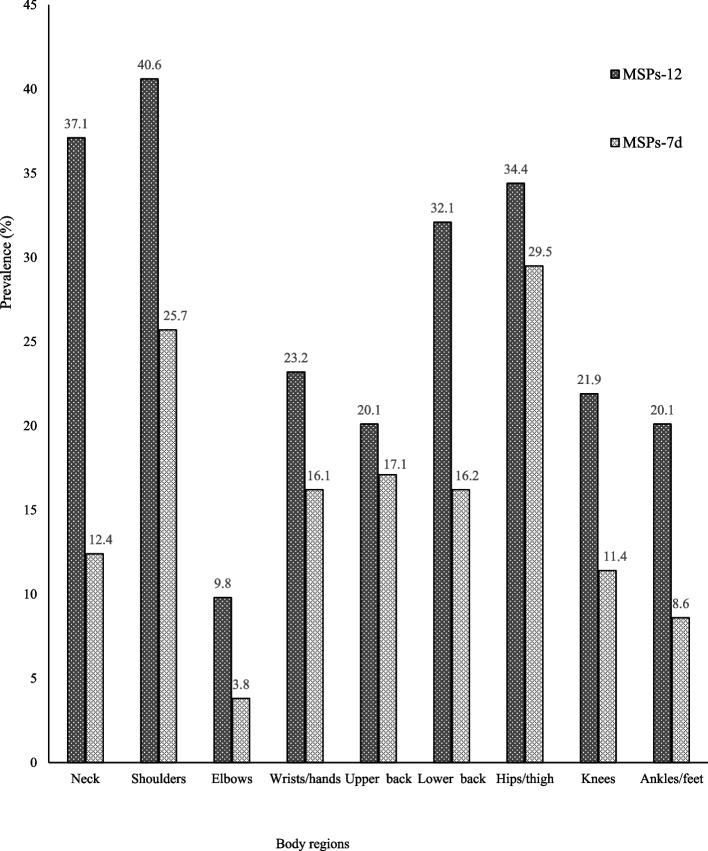
Prevalence of MSPs according to body regions. MSPs-12: prevalence of musculoskeletal pains during the 12 last months; MSPs-7d: prevalence of musculoskeletal pains during the seven last days

MSPs-12 was more frequently reported on shoulders (40.6% [95%CI.34.4–47.2%]), followed by neck (37.1% [95%CI.31.0–43.5%]), and hips/thigh (34.4% [95%CI.28.5–40.8%]) (Fig. 2). MSPs-7d were more frequent on hips/thigh (29.5% [95%CI.21.6–38.8%]), followed by shoulders (25.7% [95%CI.18.3–34.8%]), and upper back (17.2% [95%CI.11.1–25.5%]).

Proportions of MSPs-12 and MSPs-7d were significantly highest in basketball players with values of 29.5 and 30.5%, respectively. No statistically significant difference of MSPs-12 and MSPs-7d proportion was noted between athletic status (Table 2). Upon stratifying analysis by body regions, we observed that proportion of MSPs-12 was still highest in basketball players on shoulders (29.7%, *P* = 0.02), wrists/hands (34.6%, *P* = 0.001), (40.2%, *P* = 0.0002), and knees (38.8%, *P* = 0.002). In contrast, proportion of MSPs-12 was higher in tennis players on shoulders (36.4%, *P* = 0.002) (Table 2). Results were a bit contrasted for MSPs-7d, with higher proportions found on shoulders (29.6%, *P* = 0.04) for tennis players, wrists/hands (29.4%, *P* = 0.03) for basketball and football players, and hips/thigh (38.8%, *P* < 0.00001) for basketball players.

**Table 2:** Prevalence of MSD according to body region, sport discipline and athletic status

**Discipline**	MSPs -12 ( +)	Neck	Shoulders	Elbows	Wrists/hands	Upper back	Lower back	Hips/thigh	Knees	Ankles/feet
Athletics	14.7	13.3	11.0	0.0	7.7	20.0	19.4	11.7	16.3	13.3
Basket-ball	**29.5**	25.3	**29.7**	27.3	**34.6**	26.7	30.6	40.3	38.8	35.6
Football	**22.8**	27.7	20.9	13.6	23.1	24.4	16.7	18.2	10.2	26.7
Rugby	17.1	20.5	14.3	18.2	3.9	15.6	15.3	11.7	14.3	8.9
wristling	4.5	3.6	4.4	4.6	7.7	2.2	2.8	0.0	0.0	0.0
Tennis	12.5	9.6	19.8	**36.4**	23.1	11.1	15.3	18.2	20.4	15.6
***P*-value** **α**	***0.003***	*0.97*	***0.02***	***0.002***	***0.001***	*0.70*	*0.05*	***0.0002***	***0.002***	*0.1*
**Status**										
Professional	64.3	24.1	34.1	72.7	40.4	31.1	33.3	61.0	38.8	42.2
Amateur	35.7	75.9	65.9	27.3	59.6	68.9	66.7	39.0	61.2	57.8
***P*-value** **α**	***0.81***	***0.009***	*0.61*	*0.38*	*0.46*	*0.46*	*0.60*	*0.52*	*0.65*	*0.34*
**Discipline**	MSPs- 7d ( +)	Neck	Shoulders	Elbows	Wrists/hands	Upper back	Lower back	Hips/thigh	Knees	Ankles/feet
Athletics	12.4	15.4	7.4	0.0	5.9	11.1	23.5	16.1	16.7	11.1
Basket-ball	**30.5**	30.8	22.2	0.0	**29.4**	33.3	23.5	38.7	41.7	22.2
Football	19.1	15.4	22.2	75.0	**29.4**	11.1	11.8	6.5	33.3	55.6
Rugby	9.5	15.4	7.4	0.0	17.6	0.0	11.8	0.0	0.0	0.0
wristling	7.6	15.4	11.1	0.0	0.0	27.8	11.8	3.2	0.0	0.0
Tennis	**21.0**	7.7	29.6	25.0	17.6	16.7	17.7	35.5	8.3	11.1
***P*-value** **β**	**<** ***0.0001***	*0.41*	***0.04***	***0.27***	***0.03***	*0.43*	*0.26*	**<** ***0.0001***	*0.4*	*0.4*
**Status**										
Professional	64.8	*15.4*	***29.6***	***50.0***	***35.3***	*27.8*	*35.3*	***29.0***	***50.0***	*55.6*
Amateur	35.2	*84.6*	***70.4***	***50.0***	***64.7***	*72.2*	*64.7*	***71.0***	***50.0***	*44.4*
***P*-value** **α**	***0.85***	*0.11*	***0.47***	***0.52***	***0.95***	*0.45*	*0.92*	***0.39***	*0.30*	*0.21*

MSPs-12: prevalence of musculoskeletal pains during the 12 last months

MSPs-7d: prevalence of musculoskeletal pains during the seven last days

*P*-value α: comparison of MSPs-12 according to body regions

*P*-value β: comparison of MSPs-7d according to body regions

Multivariate analysis, presented in Table 3, showed a significant reduction of MSPs-12 risk by 66% in football players compared to athletics (OR = 0.34; 95% CI. 0.14—0.83; *P* = *0.01*). Again, football players had reduced risk of MSPs-12 on lower back (OR = 0.25; 95% CI. 0.10—0.63; *P* = *0.003*) and on knees (OR = 0.28; 95% CI. 0.08—0. 95; *P* = *0.04*), compared to those practicing athletics. In contrast, tennis players were more at risk of MSPs-12 on shoulders (OR = 3.14; 95% CI. 1.14–8.68; *P* = *0.02*), wrists/hands (OR = 5.18; 95% CI.1.40–11.13; *P* = *0.01*), and hips/thigh (OR = 2.90; 95% CI. 1.1–8.38; *P* = *0.04*). Professional athletes were protected from MSPs-12 on neck pain, with a significant risk reduction by 61% (OR = 0.39, 95% CI. 0.21–0.75, *P* = *0.03*).

**Table 3:** Factors associated with MSPs-12 and MSPs-7d among athletes

			Global	Neck	Shoulders	Wrists/hands	Lower back	Hips/thigh	Knees
		**Categories**	**OR (95%CI)**	**OR (95%CI)**	**OR (95%CI)**	**OR (95%CI)**	**OR (95%CI)**	**OR (95%CI)**	**OR (95%CI)**
**Overall**	MSPs-12 ( +)	Athletics	1		1	1	1	1	1
		Football	0.34 (0.14–0.83)*				0.25 (0.10–0.63)*		0.28 (0.08–0.95)*
		Tennis			3.14 (1.14–8.68)*	5.18 (1.4–19.13)*		2.90 (1.1–8.38)*	
		Amateur		1					
		Professional		0.39 (0.21–0.75)*					
	MSPs-7d( +)	Athletics	1	1		1			
		Wrestling		5.93( 1.16–11.58)*		3.60 (1.87–7.58)*			
		Tennis	3.20 (1.20–8.54)*						
**Amateur**	MSPs-12 ( +)	Athletics				1	1		1
		Football				0.10(0.01–0.96)*			0.20 (0.04–0.94)*
		Tennis				9.45 (1.20–12.16)**			
		Female					1		
		Male					2.43 (1.10–5.39)**		
	MSP-7d( +)	Athletics					1		
		Football					0.11 (0.01–0.96)*		
**Professional**	MSPs-12( +)	Athletics					1	1	
		Basket-ball						4.20 (1.18–9.47)*	
		Football					0.17 (0.04–0.74)*		

MSPs-12: prevalence of musculoskeletal pains during the 12 last months

MSPs-7d: prevalence of musculoskeletal pains during the seven last days

OR odds ratios

*P < 0.05, **P < *0.01*

Tennis players had a threefold higher overall risk of MSPs-7d (OR = 3.20; 95% CI. 1.20–8.54; *P* = *0.02*) compared to those practicing athletics. Irrespective of body region, wrestlers had a ~ fourfold higher risk of MSPs-7d in wrist/hand (OR = 3.60; 95% CI. 1.87–7.58; *P* = *0.01*) and ~ sixfold higher on shoulders (OR = 5.93; 95% CI. 1.16–11.58; *P* = *0.03*), compared to those practicing athletics.

Depending on athletic status among amateurs, odds of MSPs-12 on lower back were more than two times higher (OR = 2.43; 95% CI. 1.10–5.39; *P* = *0.02*) in males when compared to their female counterparts. Then, compared to track and field athletes, footballers were protected from MSPs-12 on lower back (OR = 0.10; 95% CI. 0.01–0.96; *P* = *0.04*) and knees (OR = 0.20; 95% CI. 0.05–0.94; *P* = *0.04*), whereas tennis players had a high risk of MSPs-7d on wrists/hands (OR = 9.45; 95% CI. 1.20–12.16; *P* = *0.003*). Risk of MSPs-7d lower back was significantly reduced by 89% in footballers (OR = 0.11; 95% CI 0.01–0.96; *P* = *0.04*) compared to those practicing athletics.

Among professional athletes, the odds of MSPs-12 on hips/thigh region were four times higher (OR = 4.20; 95% CI. 1.18–9.47; *P* = *0.02*) in basketball players, but footballers were protected by 83% from MSPs on lower back (OR = 0.17; 95% CI. 0.04–0.74*; P* = *0.01*), compared with athletic runners.

## Discussion

This study was designed to evaluate the prevalence of MSPs among Senegalese athletes according to sport disciplines and athletic status.

Overall prevalence rates of MSPs-12 and MSPs-7d were 70 and 32.8%, respectively. There are few studies assessing MSPs on all the nine body regions in different sports, most of them being focalized on one or two body regions [25–29]. These findings are not consistent with those reported by Oliveira et al. [28] respective values of 54.2 and 43.5% among Brazilian adolescent amateurs practicing multiple sport disciplines (i.e., basketball, handball, judo, swimming, volleyball). Other studies reported higher values in Germany (MSPs-12 = 81.1%, MSPs-7d = 49%) and Norway (MSPs-7d = 84%) [25, 30]. A recent study by Owoeye et al. [28] reported MSPs-12 estimate of 26% in apparently healthy collegiate practicing soccer and basketball. Moreover, Goes et al. [29] found a prevalence of pain on joints (tendinopathy) of 30.3% in elite athletes from five disciplines (rugby, soccer, combat sports, handball, and water polo).

Pain was more frequently found in males compared to females, and this is not consistent with findings from earlier reports [25, 30]. Higher rates of pain in female athletes would be related to their earlier maturity associated with hormonal discrimination [31]. Also, anatomical features in females could be additional risk factors of of pains [31]. In addition, as reported by Shan et al*.* [32] that males have a higher pain threshold than females could also justify higher rates of MSPs in females.

We reported higher rates of MSPs-12 on shoulders and hips/thigh regardless of sport discipline. Such results were reported previously in Brazil [27] in adolescents amateur athletes (basketball, handball, judo, swimming, volleyball) where authors found MSPs-12 and MSPs-7d rates on shoulders of 43.5 and 54.2%, respectively. These values are higher than 21.4% for MSPs-7d and 38.8% for MSPs-12 reported by Mohseni-Bandpei et al. [33] in Iranian athletes (swimming, rowing, wrestling, basketball, volleyball, and handball). Our findings contrast with those from several studies where MSPs-12 were predominantly seen on neck [25, 30, 34] and lower back [21, 25, 28].

Likewise, our prevalence of shoulders pain is superior to that of highlighted in an epidemiological cross-sectional study carried out among 613 Iranian overhead sports athletes in different collegiate sport fields ( swimming, rowing, wrestling, basketball, volleyball, and handball) where Mohseni-Bandpei et al. [33] reported MSPs-7d and MSPs-12 of 21.4, and 38.8% respectively. It should be noted that shoulder pain is a major performance limiting factor in aerobic and anaerobic sports requiring repeated overhead movements placed heavy loads on the dominant shoulder. In Canada, Harmath et al. [35] found MSPs-7d prevalence of 41.9% on shoulder during a study on shoulder pain and performance limitation in competitive tennis players. For some authors, joint changes in athletes are cause of acute adaptations of bones and muscles, which lead to negative impact on the range of motion of shoulders, while associated internal rotation deficits would increase the risks of shoulder pain [27, 36–39]. According to the anatomical justification, shoulder pain and injuries would be due to this internal rotation deficit, commonly kwon as ‘’glenohumeral internal rotation deficit’’ which have been recognized as joint adaptations to the practice of sports activities [39, 40]. Moreover, it is known that sport practice can lead to capsular stiffness and limit internal rotation of the glenohumeral, and thus, increase the risk of chronic pains or injuries on shoulders [37, 41].

Prevalence of MSPs varied significantly by sport disciplines, with higher values in basketball, football and tennis. Analyzing back pains in German elite athletes, Fett et al. [25] found similar results to those in the present study. Football and basketball are team sports and contact with high physiological and biomechanics demands. Both disciplines demand high level of aerobic and anaerobic capacities along with integration of physical characteristics. Frequent jumping, landing and changes in direction make up much of physical load of competitive games, which therefore, expose players to high level of eccentric muscle contractions and joints solicitations [42, 43]. Again, the intermittent aspect of basket-ball, football and tennis implies postural deviations and sudden changes in speed which are common to these three sports; and well considered as an important key factor on occurrence of pains and injuries on observed body regions [44–46]. Additional reasons of more frequent pains in basketball could be specific anthropometric demands such as height and weight which impact positively performances and increase the risk of pain and injuries [47, 48].

According to body articulations, MSPs were more frequent on shoulders, wrists/hands, hips/thigh, knees in basketball, and on elbows, shoulders in tennis. In tennis players, high prevalence of pain was found on shoulders and elbows which is in line with previous studies. Sport disciplines such as tennis, also termed as intermittent sport disciplines, imply repeated overhead motions that place important loads on dominant shoulder, and then, result in pain on this body region [49, 50]. Besides, pain on elbow is the most common cause of lateral sided in tennis, and 50% of tennis players will get an episode of follow-up-requiring pain of elbows during their career [51, 52].

The prevalence of MSPs-12 was higher in amateurs compared to that seen in professionals. This finding does not support that reported by Hoskins et al. [53] who found higher prevalence of lower back pain in Australian semi-elite athletes compared to their elite counterparts. This result could also be related to training techniques given fact that physical and psychological demands of professional athletes during competitions are thought to be higher than those of amateurs.

Multivariate logistic regression analysis revealed significant association between risk of MSP in body regions and some athletes’ characteristics (athletic status, gender, and sport discipline). Regardless of athletic status, overall risk of MSPs on shoulders, wrist/hands, and hips/thigh was higher in tennis players compared to those practicing athletics. Tennis players had higher risk of MSPs irrespective of body regions. This variability in MSPs risk is likely due to existing correlation between hyperflexion motion and incidence joints pains in athletes. Furthermore, there is a strong relationship between flexion and excessive stretching of certain musculoskeletal joints and occurrence of pain or discomfort. Thus, stress-related mechanical pressure on non-contractile tissue are sufficient to stimulate musculoskeletal pain receptors according to intermittent or continuous flexion on some joints as those of the lumbar spine [7]. To all justification, we could associate the glenohumeral internal rotation deficit on shoulders as additional explanation of this result [37, 39–41].

It is well known that MSPs incidence, prevalence, causes and risks are strongly dependent on sport modality [29, 44, 54–56]. Biomechanical and physiological demands of the musculoskeletal system following the increase in training loads, techniques in relation to athletic status in some sports disciplines before chronic adaptations could to be the main cause of pain reported within the week or in a year. Zemková et al. [57] identified that fatigue of the trunk muscles, induced by excessive loading of musculoskeletal system, is one of main sources of pain in athletes. To this regard, some studies on pain in sports have highlighted that factors, such as high training volume, repetitive motions, high physical loads, repetitive mechanical strain and extreme body(spine) positions, might be responsible for variation in risk of pain and injuries on body regions [7, 25, 58]. Nevertheless, athletes practicing discipline such as basketball, tennis and wrestling face important biomechanical, physiological demands, morphological requirements, and more positional solicitations of some joint regions of during training sessions and competitions, that can reduce or increase the risk of MSPs.

## Conclusion

In conclusion, this study pointed out high prevalence of MSPs-12 and MSPs-7d in athletes. Depending on body region, prevalence of MSPs varied significantly by sport disciplines, gender, and athletic status. Also, these three variables were associated either with decreased or increased risk of MSPs on a specific body region specific of athletes, especially in tennis and football players. The occurrence of MSPs impacted negatively performances of athletes and their health via increased exposure to injuries. There is urgent need to prevent MSPs in sub-Saharan African athletes to optimize their biomechanical performances and guarantee their wellbeing.

### Limitations

Comparability of findings from this study is limited given that prevalence of MSPs varies according to questionnaire used, most studies use self-report questionnaires with a special characterization of pain and perception. In addition, small sample sizes for some disciplines could constitute a bias. Further studies on sub-Saharan athletes should take into account age, weekly training volume, quality of life and sleep, which are important factors involved in occurrence of MSPs.

## Data Availability

Data can be shared upon contact with the correspondence author.
